# Performance and healthcare team processes and structure impact player availability in professional men’s football

**DOI:** 10.1136/bmjsem-2025-002664

**Published:** 2025-07-18

**Authors:** Kunle Odetoyinbo, Carly D McKay

**Affiliations:** 1 Premier Game Academy Auditing Company, Premier League, London, UK; 2 Department for Health, University of Bath, Bath, UK; 3 UK Collaborating Centre on Injury and Illness Prevention in Sport, University of Bath, Bath, UK

**Keywords:** Football, Sports & exercise medicine, Injury, Effectiveness

## Abstract

**Objectives:**

To determine whether the structures and processes of an English Championship football club’s performance and healthcare team (PHCT) were associated with player availability (PA) during periods of match congestion (≤3 days between matches).

**Methods:**

This sequential explanatory mixed-method case study included 10 practitioners from the PHCT. Participants completed team process/structure questionnaires two times per month during the 2017–2018 season. PA and match frequency data were provided by the PHCT, who also participated in a postseason focus group. Associations between PA and team structures/processes were assessed using Pearson correlations. Framework analysis was used to explore PHCT perceptions of teamwork effectiveness. Results synthesis was guided by the Integrated Team Effectiveness Model.

**Results:**

Mean PA during match congestion was 78.1% (95% bias-corrected and accelerated bootstrap CI (BCa): 76.2%, 80.4%) compared with 84.2% (95% BCa: 80.6%, 87.3%) during uncongested periods. There were significant associations between match frequency and PA (r= −0.68; 95% BCa: 0.32, 0.93; p=0.008) and PHCT processes and PA (r=0.53; 95% BCa: 0.09, 0.89; p=0.035). Having more PHCT meetings (r=0.46; BCa 95%: 0.22, 0.82; p=0.048) and greater satisfaction with those meetings (r=0.41; BCa 95%: 0.04, 0.07; p=0.043) were associated with higher PA, irrespective of match frequency. During match congestion, the PHCT reported that resource and task coordination issues negatively impacted their processes.

**Conclusions:**

Match congestion was associated with disruptions to PHCT processes and structure, with negative implications for PA.

**WHAT IS ALREADY KNOWN ON THIS TOPIC**

- Fixture congestion in football is linked to a higher incidence of injuries. It may be associated with workload, the quality of internal communication among staff and the head coach’s leadership style.
- Multidisciplinary teams often underperform without adequate attention to their processes and structures.

**WHAT THIS STUDY ADDS**

- The Integrated Team Effectiveness Model (ITEM) is an effective framework for studying performance and healthcare teams (PHCTs) in professional football.
- Match frequency affects player availability (PA) and is linked to the PHCT structure and processes.

**HOW THIS STUDY MIGHT AFFECT RESEARCH, PRACTICE OR POLICY**

- This is the first study to demonstrate the utility of the ITEM for studying teamwork effectiveness in professional football; its methods provide a foundation for interprofessional teamwork research in sports.
- To optimise PA, PHCT should strategically prioritise team meetings, foster an interprofessional teamwork approach, ensure adequate resources and maintain alignment with coaching staff.

## Introduction

 Elite men’s football presents significant performance and health challenges due to the high risk of injury^1^ and frequency of competition.^2 3^ Football squads with fewer injuries and consistent, uninterrupted training are likely to finish in higher league positions.^4^ Consequently, an increasing range of practitioners are employed within multidisciplinary performance and healthcare teams (PHCTs). While PHCTs have benefited from considerable scientific advances informing preparation, participation and recovery from training and competition, limited evidence is available on the effectiveness of PHCT work and their teamwork approach. Although the importance of collaboration and interprofessional education to patient outcomes and team function is increasingly recognised within sports science and medicine,^5 6^ their structured implementation in sport and exercise medicine remains limited.

‘Teamwork’ within multidisciplinary teams refers to the degree to which behavioural processes (eg, communication, collaboration, expertise sharing) or non-technical skills are used to accomplish interdependent work and/or the affective, cognitive and motivational states (eg, cohesion or conflict) that emerge during that work.^7 8^ Organisational research outside of sport that includes healthcare, aviation and manufacturing,^7 9 10^ as well as teamwork studies employing theoretically guided framework models,^11^ highlights that multidisciplinary teams working in highly pressured environments often have suboptimal and sometimes catastrophic outcomes without due consideration of their processes and structure.^12^ Organisational studies suggest that multidisciplinary teams that interact collaboratively and participate in shared decision-making (defined as an interprofessional teamwork approach) have favourable outcomes when compared with teams whose individuals work towards independent goals with limited interaction or shared decision-making (ie, a multidisciplinary teamwork approach).^13^

The relevance of teamwork effectiveness, reflecting relationships between a PHCT’s structure and processes in elite football, remains largely unknown. UEFA’s ongoing elite club injury research^14 15^ indicates that PHCTs with higher communication quality experience lower injury burden and higher player availability (PA). UEFA’s prospective studies implicate match intensity and communication as risk factors for injury,^16^ consistent with the evidence from other multidisciplinary teams working under time constraints and pressure.^17^ Furthermore, research focused on interprofessional collaboration between sport science and medical professionals has indicated potential relationships with care quality.^18^ Research on PHCTs and outcomes of their teamwork effectiveness is therefore warranted due to potential implications for professional football players’ performance and health. This study investigated whether the structures and processes adopted by a PHCT were associated with PA during periods of match congestion, using the Integrated Team Effectiveness Model (ITEM).^11^

## Materials and methods

This sequential explanatory mixed-method case study was conducted throughout the 2017–2018 English Championship football season.

The processes adopted by a PHCT, their structure and outcomes of their work were investigated using the ITEM to establish their teamwork effectiveness. The ITEM, a flexible framework adaptable to specific teams and settings,^19^ was used (box 1) to investigate a PHCT in a single club. Teamwork was considered a linked process: *inputs* (team composition, characteristics, ie, structure), translated through *mediators* (ie, team member interactions and processes), to *outputs* (ie, outcomes of team activities). It is implicit that a team’s processes can be investigated by considering the interactions of its members, given the potential for complex team dynamics.^20 21^

Box 1 **The adapted Integrated Team Effectiveness Model framework that was used to guide this study**

**Team structure (inputs)**

Task type: management and delivery of performance and health

Task features: interdependence/autonomy, specialised knowledge/expertise

Team composition: disciplines, size, diversity, tenure, team premise

Organisational context: external support

**Team processes (mediators)**

Team meetings: communication, collaboration, coordination, decision-making, participation, conflict, evaluation, goals/objectives

**Teamwork effectiveness (output)**

Objective: player availability

Subjective: perceived effectiveness

### PHCT and data collection

10 practitioners delivering performance/health support to professional first team players within a club were recruited, comprising sports science and medicine staff (ie, the PHCT) (table 1). Each practitioner completed the team process questionnaire every 2 weeks (14 times in-season, from October to May) and a team structure questionnaire 7 days post season. 1 week later, they participated in a focus group discussion.

**Table 1:** Participant characteristics

Role	Subgroup	Staff (n)	Tenure within the club (years)	Tenure range (years)	Leadership role
Sport medicine staff	Physiotherapists	3	5.5	4.2–5.8	No
	Head of Sport Medicine	1	2.1	N/A	Yes
	Soft tissue specialist	1	0.7	N/A	No
Sport science staff	Exercise scientists (including Head of Sport Science)	2	3.9	3.6–5.6	Yes (1)
	Strength and conditioning specialist	1	2.6	N/A	No
	Data analysts	2	4.1	2.6–5.5	No

### Team Process Questionnaire

Two times per month during the competitive season, all PHCT staff completed a validated team process questionnaire^20^ (online supplemental content). Part 1 assessed practitioner participation, interaction and satisfaction with meeting discussions and results, using a five-point Likert scale ranging from ‘not at all’ (score=1) to ‘a very great extent’ (score=5). It also identified the PHCT ‘teamwork approach’ (ie, interprofessional or multidisciplinary).^20 22^

Part 2 of this questionnaire assessed PHCT evaluations (work audits/reviews), the frequency of staff informal interactions (outside of meetings), team goals and objectives, individual influence on decisions, the match frequency’s impact on work performed and the frequency of team meetings, in line with the processes identified within the adapted ITEM. Questionnaires were returned within 10 days of receipt to minimise recall error.

### Team Structure Questionnaire

Each PHCT completed a bespoke team structure questionnaire 7 days post season, to capture organisational context, task types/features and staffing composition (online supplemental content). Guided by the adapted ITEM, the questionnaire included open and closed Likert scale questions (‘not at all’ (score=1) to ‘a very great extent’ (score=5)) to describe the PHCT structure. Triangulation through a focus group discussion was used to enhance the content validity of this questionnaire and increase confidence in the findings.^23^ The 7-day gap post season allowed practitioners to reflect before completing the questionnaire.

### Focus group discussion

PHCT members participated in an audiorecorded, in-person focus group discussion to explore collective understanding of their work and the environment in which it was conducted. Moderated by the lead researcher and supported by a facilitator (research assistant) who observed and took notes, the discussion followed a semistructured guide based on the adapted ITEM and questionnaire results, aligning with an explanatory mixed-method design. The lead researcher transcribed the audio recording verbatim, and the research assistant verified accuracy. Observational notes were added to support analysis.

### Match frequency

Match congestion was operationalised based on match frequency. Based on the team schedule and using both the number of hours between consecutive matches and the number of matches played (eg, over 6.9 days/3 matches played=match frequency of 2.3), match frequency illustrated the time frames within which the PHCT could conduct their work for given stages of the season. ‘Match congestion’ for any given period was identified when there was a match frequency of ≤3 days.

### Player availability (PA)

PA data, provided monthly throughout the study by the head of sports medicine, were correlated with team process questionnaire responses collected within the same month. These data only included players deemed eligible to compete in each match as determined by the PHCT and included players omitted for technical or rule violations who were not considered ill or injured.

### Data analysis

Quantitative and qualitative data were first analysed separately and subsequently merged to draw inferences across both, including how PHCT structure might further inform an understanding of relationships between processes and outcomes. This approach explored convergence or contradictions in findings, consistent with an explanatory mixed-method design.^24^

### Quantitative analysis

Descriptive statistics are presented as the mean±SD for the team process questionnaire. Data were checked for normality using the Shapiro-Wilk test, and linearity and homoscedasticity were evaluated using box plots and scatterplots. Questionnaire domain scores were averaged per biweekly ‘stage’ to calculate overall scores. Bivariate Pearson correlation assessed relationships between PHCT process scores, PA, match frequency and congestion for the 14 biweekly stages. A separate Pearson correlation analysis was conducted to examine the relationship between the PHCT’s ‘teamwork approach’ and PA across the season. Due to the small sample size (n=14), all correlations used the bootstrap method to generate bias-corrected and accelerated bootstrap CI (BCa). SPSS Statistics for Windows V.23.0 was used for analysis.

Descriptive statistics extracted from the team structure questionnaire highlighted PHCT characteristics, including organisational support for innovation, practitioner specialist roles and their integration with the team (0–100% scale).

### Qualitative analysis

Open-ended responses from the team structure questionnaire were analysed using deductive content analysis, involving familiarisation, formulating meaning units and codes and developing categories and themes.^25^ The ITEM guided theme identification, though new themes were allowed to emerge.

Focus group data were assessed using framework analysis,^26^ which involves familiarisation, identifying themes and categories, developing a coding index and assigning data to categories. This process supported the creation of descriptive and subsequently explanatory accounts, which were synthesised with the quantitative data for interpretation.

To ensure that responses were accurately reflected, the qualitative analysis adhered closely to participant statements, acknowledging the lead researchers’ practitioner background. The research assistant verified that interpretations, categorisations and themes were consistent with the data to enhance analytical rigour.

### Patient and public involvement

Members of the PHCT, athletes and coaches were not involved in setting the research question or determining the outcomes. Still, PHCT members played a key role in managing the study by facilitating data collection and transfer. The main results were shared with participants, and their input will inform the selection of an appropriate knowledge exchange method within the football community.

## Results

### PA and match frequency

**Figure 1:**
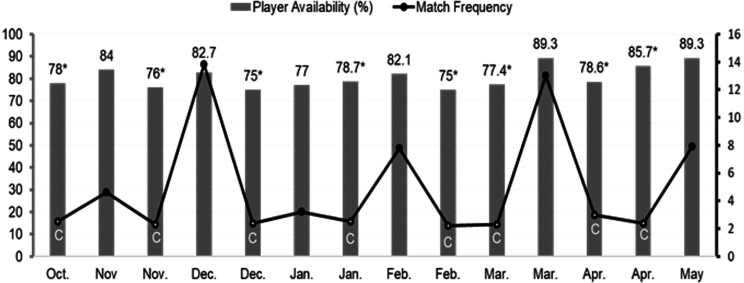
Player availability and match frequency in-season 2017–2018. Note: match frequency is represented by the number of days between successive matches where match congestion is symbolised with ‘C’ to illustrate when match frequency ≤3 days (ie, match congested stages of the season). Player availability = per cent of total squad available for match selection, where * corresponds with match congestion.

PA over the course of 39 matches averaged 80.6±4.9% (range 75–89%), during which the PHCT had an average of 5 days (SD=4, range 2–14) between matches to conduct their work. Match congestion was reported during 16 of 28 weeks, during which 78.1±3.2% of the playing squad were available for selection compared with 84.2±4.7 outside of these periods. When matches were more congested, PA was reduced for competition (r= −0.68; 95% BCa (0.324, 0.929) p=0.008) (figure 1).

### Team processes and PA

PHCT responses regarding team processes appear in table 2. The processes adopted by the PHCT in meetings varied during the season and were positively associated with PA (r=0.53; 95% BCa: 0.087, 0.888 p=0.035). The number of meetings (r=0.46; 95% BCa: 0.219, 0.821; p=0.048), PHCT members’ satisfaction with results of those meetings (r=0.41; 95% BCa: 0.042, 0.714 p=0.043) and audit/evaluation of their work (r=0.44; 95% BCa: 0.374, 0.878 p=0.009) were also associated with PA. When PHCT members perceived there to be negative interactions within meetings, PA was lower (r= −0.57; 95% BCa: −0.087, −0.097 p=0.03). The frequency of interactions and contacts between team members outside of meetings did not have any statistically significant association with PA (r=0.1; BCa −0.621 to –0.737) or match frequency (r=−0.03; BCa −0.805, 0.677) over the course of the season.

**Table 2:** Average team process questionnaire domain responses across the season

Domain	Mean score	Range
Negative interaction	1.9	1–2
Result satisfaction	3.5	3–4
Process satisfaction	3.8	3–5
Personal participation	3.4	3–4
Goals/objectives	3.8	3–4
Audit/evaluation	2.7	2–3
Number of meetings per 2-week reporting period	6.5	5–10
Frequency of informal exchanges per 2-week reporting period	3.7	3–4
Influence on team decisions	3.3	2–4
Impact of match frequency	2.6	2–3

Likert scale questions ranging from 1 (‘not at all’) to 5 (‘a very great extent’).

### Team structure

PHCT areas of practice included training load monitoring, recovery activities and injury treatment, all of which were reported most frequently during congested periods. Practitioners reported that specialist knowledge accounted for 82±8% of their total work. Injury treatment and rehabilitation were primarily managed by sports medicine staff, while sports science staff focused on training load and recovery monitoring. All members of the PHCT conducted data management and exercise prescription. 80% of the PHCT perceived their work approach as predominantly interprofessional rather than multidisciplinary.

### Match frequency, injuries and PHCT structure

Table 4 illustrates the primary themes from the Team Structure Questionnaire. Altered working arrangements during match-congested periods, driven by increased injuries, were cited by 70% of the PHCT. Furthermore, resource issues, including staffing and equipment, were considered to impact engagement with other staff, as well as a need to prioritise decisions based on data during busy periods. These results converge with the fluctuations in the teamwork approach reported in the quantitative data across varying match frequencies.

**Table 3:** Thematic analysis of open-ended questions in the Team Structure Questionnaire

Example codes	Categories	ITEM theme
Working on own more Working more independently Less time with colleagues Less staff time together	Independent practice	Task featuresInterdependenceAutonomy
Increased workload Increased injuries/travel Reduced time with players Less time to respond Increased work stress	Task load and demands	Task featuresWork cycle
Structure consistent Team stays the same Infrastructure constant Same team working	Team structure	Team designWork context

### Focus group discussion

10 themes and 7 core concepts emerged relating to PHCT structure and processes (table 4). Match congestion was associated with more injuries, necessitating specific skills (‘contextual competencies’) when working with limited resources. The PHCT perceived that these resources had to be spread thinly between injured players and those available to train and compete. Despite the evolving refinement of ‘teamwork practices’, resource constraints reduced time for meetings and collaboration, despite these being considered pivotal for reviewing prior work and planning future activities. These challenges were perceived to negatively impact PA, aligning with the quantitative findings (ie, the association between match frequency, number of meetings and PA).

**Table 4:** Coding index and core concept development from the focus group discussion

Themes	Core concepts	ITEM framework component
Professional specialisation	Contextual competency	Teamwork structure
Work cycle	Resource workload capacity	
Interprofessional collaboration	Structured interprofessional collaboration	Teamwork process
Directed teamwork		
Team social traits	Cohesiveness	
Team composition	Structured interdependency	
Teamwork-reliant practice		
Performance health judgements	Interprofessional decision-making	
Squad management objectives	Performance management outcomes/evaluation	Teamwork outcomes
Evaluated teamwork effectiveness		

Pressure to ‘win the next match’, particularly when matches were congested and results were not favourable, was also reported. This coincided with PHCT staff citing instances where the head coach overrode their ‘return to play’ decision-making, highlighting the challenges in their work.

## Discussion

Meetings were pivotal for integrating knowledge across disciplines, aligning well with practitioners’ perception of satisfaction regarding discussions, solutions that emerged and their individual participation in meetings over the season. This may underpin the strong association between the number of meetings and PA in this study, as illustrated by the positive correlation between practitioners’ ratings of their confidence in and responsibility for plans that emerged from meetings (‘results satisfaction’) and PA. However, match congestion led to a reduction in meeting frequency, resulting in a shift toward autonomous work and associated lower productivity. Similar trends have been observed in other high-pressure healthcare settings.^27^

Consistent with the findings from match congestion research,^3^ match frequency was strongly associated with PA. During congested periods, the PHCT faced challenges (ie, more injuries, less preparation time, limited resources), leading to prioritisation of practices, including recovery and risk assessments. PHCT practitioner interactions in meetings were strongly associated with PA. While teamwork scores could not definitively distinguish interprofessional from multidisciplinary approaches at specific time points, seasonal variations indicated that the PHCT meetings were conducted on a continuum between the two.

An interprofessional approach involved greater collaboration and shared decision-making between practitioners, evident when matches were not congested; however, when matches were congested and results were not favourable, practitioners cited higher levels of stress resulting from the need to ‘win the next game’ and coaching staff interventions. This resulted in deviations from collective decisions and alterations to processes, which practitioners believed contributed to additional injuries.^28^ This echoes findings from sport science and medical team research where interprofessional collaboration has been considered important to care delivery, suggesting that these benefits may be diminished during fixture congestion, when collaborative practices are most constrained.^18^ The need to win is inherently the driving force behind PHCT work, though this may conflict with their need to protect the long-term health of players. Relationships between the PHCT and coaching staff (the ‘organisational context’ in ITEM^11^) are an important consideration and may have contributed to PA in this study.

English Premier League practitioners have noted that in-season stressors negatively affect their work.^25^ Practitioners worked more autonomously under high workloads, reducing shared decision-making. Similar trends, linked to high pressure and stress, resource scarcity, and individual goal pursuit, have been observed in healthcare and European football teams.^29 30^ This shift to a multi-disciplinary approach and reduced meetings was associated with lower PA. The change to autonomous working represents an ITEM structural alteration, adding further insight to the relationships between PHCT processes and PA. For instance, informal communication (eg, ‘corridor conversations’) was hypothesised to support teamwork in this context, but showed no relationship with PA. This contrasts with medical settings, where it explains up to one-third of team performance variance.^31^ Despite spending long workweeks together, competition demands and travel often split the PHCT in this study, suggesting unmeasured communication types may have occurred that have not been considered.

The emergent theme of ‘structured interdependency’ highlights the importance of PHCT members collaborating across disciplinary boundaries, rather than focusing solely on their own expertise. However, during periods of match congestion, this approach became challenging, leading to a reversion to discipline-specific work that negatively impacted teamwork effectiveness, consistent with findings from other healthcare-related studies.^32^ This link between PHCT structure (staff disciplines and interdependency) and processes (collaboration and communication) provides a novel perspective on teamwork effectiveness and its relationship with PA.

Practitioner responses in this study indicated a limited amount of audit or evaluation of PHCT work; however, it was more frequently conducted outside of congested matches and was then positively associated with the number of players available for competition. Learning through feedback is evidenced as central to the effectiveness of teamwork in care settings,^33^ supporting the idea that PA improves when PHCT practitioners collaborate and consistently audit and learn from their work.^6^

### Clinical implications

There is evidence that PHCT communication is associated with injury burden and PA in elite-level football,^12 13^ and the current study highlights the importance of optimising PHCT structure and processes to support communication quality. PHCTs should therefore prioritise team meetings, foster an interprofessional teamwork approach, ensure adequate resources for auditing and evaluating their work and try to maintain alignment with coaching staff. These actions are particularly important during periods of fixture congestion.

### Limitations

The findings of this case study may be limited by the specific circumstances of a single football club, with unique antecedent conditions, which restricts its explanatory range and external validity. Additionally, the small sample size of practitioners providing repeated data limited study power such that predictive analyses could not be reliably conducted. The consequent correlational approach constrains causal inferences, and therefore, the directionality of the relationships under investigation should be interpreted with caution.

Although validated team process measures were employed, bespoke elements in the team structure and part two of the team process questionnaire were not comprehensively validated, which may have introduced measurement error. However, the subsequent triangulation with focus group data may further reinforce the robustness and reliability of the findings.^22^ The study did not assess the effectiveness of actions decided in meetings, which undoubtedly impacted PA over the course of the season. Moreover, expectancy and confirmation biases may have led to an overestimation of positive team processes and structures, limiting the generalisability of the findings.

We acknowledge that this study focused specifically on a men’s football setting due to our access within the participating football club. Given the under-representation of female/woman/girl participants in sport injury research, we recognise this as a limitation and strongly recommend similar research be conducted in the women’s football context,^34^ which differs significantly from the men’s and would benefit equally from knowledge about the influence of PHCT structure and processes on PA.

## Conclusion

This novel study found that PHCT processes during varying match frequencies were associated with PA. PHCT structure, as defined by ITEM, further informed an understanding of the relationship between team processes and PA during periods where the organisational context and resources (structural inputs) altered PHCT processes. This suggests that the PHCT structure can only be translated into optimised PA if team processes are effective and resources are sufficient during fluctuating match frequencies. During match congestion, efficient information flow between staff and an interprofessional teamwork approach within meetings is associated with improved PA.

## Supplementary material

10.1136/bmjsem-2025-002664 online supplemental file 1

## Data Availability

No data are available.
