# Supplementary material for: Performance and healthcare team processes and structure impact player availability in professional men’s football
Source: BMJ Open Sport Exerc Med. 2025 Jul 18;11(3):e002664. doi: 10.1136/bmjsem-2025-002664 (PMC12278155; doi:10.1136/bmjsem-2025-002664)
Supplement: online supplemental file 1 [file bmjsem-11-3-s001.pdf]

Team Process Questionnaire

**Example: Has the frequency of matches in any way impacted your work ? (you may mark the apppropriate circle)**	1. not at all	2. to a little extent	3. to some extent	4. to a great extent	5. to a very great extent

**Team Meetings: Performance health care team meeting plans to optimise performance health. (*Personal Participation: the extent to which individuals participated in discussions*)**	1. not at all	2. to a little extent	3. to some extent	4. to a great extent	5. to a very great extent
1. Did you make suggestions about the way in which performance and or health related plans can be accomplished?					
2. Did you ask for suggestions from other performance health care team participants?					
3. Did you provide information about the situation and opportunities of players ?					
4. Did you ask others about their ideas and opinions?					
5. How much attention to and interest in the contributions of other performance health care team participants did you show?					

**Team Meetings** **(*Social Behaviour: the extent to which individuals behaved negatively towards one another*)**	not at all	to a little extent	to some extent	to a great extent	to a very great extent
6. Did others express a negative opinion about your behavior?					
7. Did you reject other people's opinions or suggestions?					
8. Did others reject your opinions or suggestions?					
9. Did you express negative opinions about anyone's behaviour?					
10. Did you feel frustrated or tense about other people's behaviour?					

**Team Meetings (*Result Satisfaction: The amount of satisfaction concerning the solutions which resulted from the discussions*)**	not at all	to a little extent	to some extent	to a great extent	to a very great extent
11. To what extent are you confident that the performance healthcare plans were appropriate ?					
12. To what extent are you committed to the performnce healthcare plan?					
13. To what extent did the final overall plans reflect your contributions?					
14. To what extent do you feel personally responsible for the appropriateness of the performance healthcare plans?					
15. How satisfied are you with the quality of the performance health care plans?	very dissatisfied	somewhat dissatisfied	neither satisfied Nor dissatisfied	Somewhat satisfied	Very satisfied

**Team Meetings** **(Process Satisfaction: the amount of satisfaction concerning the discussion process)**	Inefficient				Efficient
16. How would you describe the team meetings during this period?	1	2	3	4	5
inefficient-efficient	Inefficient				Efficient
Uncoordinated-Coordinated	Uncoordinated				Coordinated
Unfair-Fair	Unfair				Fair
Confusing-Understandable	Confusing				Understandable
Dissatisfying- Satisfying	Dissatisfying				Satisfying

**Performance Health Care Team Goals/Objectives**	not at all	to a little extent	to some extent	to a great extent	to a very great extent
17. To what extent did you achieve your goals during this period?					
18. To what extent did the performance healthcare team have shared performance health objectives during this period?					
19. To what extent did the members of the team pull in the same direction during this period?					
20. Were the performance healthcare team objectives clear during this period?					
21. Would it be fair to say that the main objective of the performance healthcare team is to optimise player availability for competition during this period?					

**Performance Healthcare Team Audit (Evaluation Of Performance)**	not at all	to a little extent	to some extent	to a great extent	to a very great extent
22. To what extent was the teams' work evaluated during this period?					
23. Did any of the PHCT provide feedback regarding your contributions during this period?					
24. To what extent has your work been innovated/changed during this period as a result of feedback and or evaluation?					

**Additional Single Items**	not at all	to a little extent	to some extent	to a great extent	to a very great extent
28. During this period how many meetings did you attend ? (state number only)	…………				
29. Was there regular contact among PHCT members during this period?					
30. Did you have *frequent informal and mutual exchanges* with PHCT members to discuss performance/health matters?					
31. Did you feel you had influence on team decisions during this period?					
32. During this period did the frequency of matches impact any of the following;					
a. Frequency communications and information exchange with other team members outside of formal meetings?					
b. Coordination of activities that involve other team members outside of formal meetings?					
c. The decision making process between performance health care team members?					
d. Collaboration with other team members?					

Definitions: **Multidisciplinary team** (professionals working together towards their own goals): **Interdisciplinary team** (professionals working together predominantly towards shared goals)

Team Structure Questionnaire: Department______________________________

The following questionnaire seeks to gather information relating to the structures that have been adopted by the performance health care team over the course of the season. Please complete each section of the questionnaire fully. If you are unsure of any aspect of the questionnaire please refer this to a member of the research team. Your answers are strictly confidential. The questionnaire should take approximately mins to complete.

Q. No.	Questions	Guide	Answer/Response	ITEM components that each question addresses
Q.1	What has been your official position and title this season?	State		Team Composition
Q.2	Were you employed full or part time?	Tick box as appropriate	FT ☐ PT ☐	
Q.3	How long have you been in your current role?	State	Years ☐ Months ☐	
Q.4	Do you consider yourself a member of a support team responsible for the performance and health?	Tick box as appropriate	Yes ☐ No ☐	
Q.5	Which discipline do you represent? e.g. physiotherapy	State		
Q.6	Approximately what % of your role requires specialist knowledge/expertise that contributes to the performance health of players?	Circle as Appropriate	0 10 20 30 40 50 60 70 80 90 100	Task Features
Q.7	Did any of the tasks you performed require the use of any particular rules/guidelines or procedures?	Tick as appropriate	Yes ☐ No ☐<br>If yes please briefly describe below.	
Q.8	Would you describe your job/tasks as predominantly interdependent or autonomous in its delivery?	Tick box as appropriate and briefly describe	Interdependent ☐<br>Autonomous ☐	
Q.9	Where are you situated geographically on a daily basis in performing your tasks relative to most other team members?	Tick a box most appropriate	In very close proximity ☐<br>Quite close ☐<br>Quite far away ☐<br>Isolated far away ☐	Team Premise
Q.10	What roles do you play in optimising the performance health of the football squad? e.g. strength and conditioning.	Briefly Describe		Task Features
Q.11	Would you consider the performance health care team to be structured as an interdisciplinary or a multidisciplinary team to meet its objectives? (See definitions below).	Tick box as appropriate and add further comment	Inter-disciplinary | Multi-disciplinary | Changeable depending upon circumstance | Neither | Comment	
Q.12	With which discipline of staff do you collaborate and interact most with in your work and why? e.g. massage therapists	Please State and describe reason(s)		
Q.13	To what degree does the wider organisation outside of the performance and healthcare team support innovation and change?	Circle as appropriate	none at all — to some extent — to a very great extent<br>0 10 20 30 40 50 60 70 80 90 100	Organisational Support
Q.14	What roles have you played in player availability decisions over the course of the season?	Briefly Describe		Task Features
Q.15	To what extent has your functional area been integrated into the performance healthcare team activities?	Circle as appropriate	not at all — to some extent — to a very great extent<br>0 10 20 30 40 50 60 70 80 90 100	
Q.16	In your role during the season did you report to someone in the team?	If yes please state	Yes ☐ No ☐ *State here*	
Q.17	Do you have a structure or procedure for resolving conflict within the performance healthcare team?	Briefly Describe		Conflict/ Cohesion
Q.18	Does the organisational structure outside of the performance health care team support further training and technical assistance?	If yes please state	Yes ☐ No ☐ *State here*	Organisational Context
Q.19	How do you influence player availability decisions during the season?	Please state		Task Features
Q.20	Does the frequency of matches have any impact on the structure of the performance health care team over the course of a competitive season?	Briefly Describe		
